# Guidelenines in the management of obstructing cancer of the left colon: consensus conference of the world society of emergency surgery (WSES) and peritoneum and surgery (PnS) society

**DOI:** 10.1186/1749-7922-5-29

**Published:** 2010-12-28

**Authors:** Luca Ansaloni, Roland E Andersson, Franco Bazzoli, Fausto Catena, Vincenzo Cennamo, Salomone Di Saverio, Lorenzo Fuccio, Hans Jeekel, Ari Leppäniemi, Ernest Moore, Antonio D Pinna, Michele Pisano, Alessandro Repici, Paul H Sugarbaker, Jean-Jaques Tuech

**Affiliations:** 1 1st Unit of General Surgery, Ospedali Riuniti di Bergamo, Italy; 2 Department of Surgery, Linköping University Hospital, Sweden; 3 Department of Internal Medicine and Gastroenterology, University of Bologna, Italy; 4 Unit of General, Emergency and Transplant Surgery, St Orsola-Malpighi University Hospital, Bologna, Italy; 5 Acute Care and Trauma Surgery Unit, Maggiore Hospital Trauma Center, Bologna, Italy; 6 Department of Surgery, ZNA Middelheim, Antwerp, Belgium; 7 Department of Surgery, Helsinki University Hospital, Helnsiki, Finland; 8 Department of Surgery, Denver Health Medical Center, University of Colorado Denver, CO, USA; 9 Department of Gastroenterology, Digestive Endoscopy Unit, IRCCS Istituto Clinico Humanitas, Milano, Italy; 10 The Washington Cancer Institute, Washington Hospital Center; 11 Department of Digestive Surgery, Rouen University Hospital, Rouen, France

## Abstract

**Background:**

Obstructive left colon carcinoma (OLCC) is a challenging matter in terms of obstruction release as well of oncological issues. Several options are available and no guidelines are established. The paper aims to generate evidenced based recommendations on management of OLCC.

**Methods:**

The PubMed and Cochrane Library databases were queried for publications focusing on OLCC published prior to April 2010. A extensive retrieval, analyses, and grading of the literature was undertaken. The findings of the research were presented and largely discussed among panellist and audience at the Consensus Conference of the World Society of Emergency Surgery (WSES) and Peritoneum and Surgery (PnS) Society held in Bologna July 2010. Comparisons of techniques are presented and final committee recommendation are enounced.

**Results:**

Hartmann's procedure should be preferred to loop colostomy (Grade 2B). Hartmann's procedure offers no survival benefit compared to segmental colonic resection with primary anastomosis (Grade 2C+); Hartmann's procedure should be considered in patients with high surgical risk (Grade 2C). Total colectomy and segmental colectomy with intraoperative colonic irrigation are associated with same mortality/morbidity, however total colectomy is associated with higher rates impaired bowel function (Grade 1A). Segmental resection and primary anastomosis either with manual decompression or intraoperative colonic irrigation are associated with same mortality/morbidity rate (Grade 1A). In palliation stent placement is associated with similar mortality/morbidity rates and shorter hospital stay (Grade 2B). Stents as a bridge to surgery seems associated with lower mortality rate, shorter hospital stay, and a lower colostomy formation rate (Grade 1B).

**Conclusions:**

Loop colostomy and staged procedure should be adopted in case of dramatic scenario, when neoadjuvant therapy could be expected. Hartmann's procedure should be performed in case of high risk of anastomotic dehiscence. Subtotal and total colectomy should be attempted when cecal perforation or in case of synchronous colonic neoplasm. Primary resection and anastomosis with manual decompression seems the procedure of choice. Colonic stents represent the best option when skills are available. The literature power is relatively poor and the existing RCT are often not sufficiently robust in design thus, among 6 possible treatment modalities, only 2 reached the Grade A.

## Background

The majority of cases of acute colonic obstruction is secondary to colorectal cancer. Up to 20% of patients with colonic cancer present with symptoms of acute obstruction [1-4]. Emergency surgery for acute colonic obstruction is associated with a significant risk of mortality and morbidity and with a high percentage of stoma creation (either temporary or permanent)[1,2,5,6]. Whereas right-sided colonic obstructions are usually treated by one-stage resection with primary anastomosis for all patients but the frailest [1], controversy continues to revolve around emergency management of obstructed left colon cancer (OLCC).

**Figure 1:**
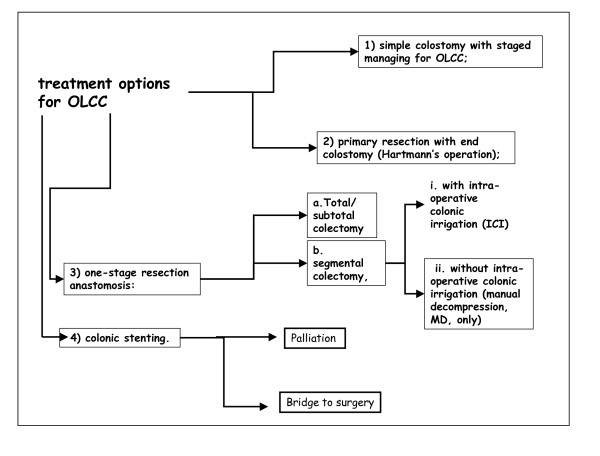
**Treatment Options for OLCC**.

Indeed several options for OLCC are available (Figure 1):

1) loop colostomy (C) or loop ileostomy and subsequent resection (2 or 3 staged procedure)

2) primary resection with end colostomy: Hartmann's procedure (HP);

3) primary resection and anastomosis (PRA):

a. total/subtotal colectomy (TC)

b. segmental colectomy, (SC)

i. with intra-operative colonic irrigation (ICI)

ii. with manual decompression (MD)

4) endoscopic colonic stenting by self-expanding metallic stents (SEMS):

a. palliation

b. bridge to surgery

The consensus conference aimed to evaluate available literature to generate evidenced based recommendations on management of OLCC. It must be stated, in advance, that suggestions coming from this study are not substitute of the clinical judgement.

## Methods

The PubMed and Cochrane Library databases were queried for publications focusing on OLCC published prior to April 2010. The following Mesh headings were used: 'colonic neoplasm', 'intestinal obstruction', 'stents', 'colectomy'. Also, text terms were used in combination such as: 'colonic obstruction', 'colonic stents', 'Hartmann's operation', 'colonic irrigation', 'colostomy', 'anastomosis'. There was no language restriction. The 'Related Articles' function in PubMed was used and the references of the retrieved articles were reviewed. Initially the Chairman (AL) and the committee members (BF, CV, LA, RA, TJJ) collaborated to the preparation of a draft inclusive of preliminary statements. Subsequently, the Chairman, the committee members and world renowned experts in the field met for a consensus conference on OLCC during the 1^st ^World Congress of World Society of Emergency Surgery and the IX Meeting of Peritoneum and Surgery (PnS) Society (Bologna, Italy, July 2010). During the consensus conference each committee member presented a summary of evidence available for each of the treatment options outlined in Figure 1. The data available from literature review were analyzed and graded according to the level of evidence validated by the American College of Chest Physicians (ACCP) systems (Table 1) [7,8]. Those presentations served to launch a discussion on optimal management of OLCC. Following exhaustive discussion the panel was asked to agree on final recommendations.

**Table 1:** Grades of Recommendations according to the American College of Chest Physicians (ACCP) 78

Grade of recommendation	Clarity of risk/benefit	Methodological strength of supporting evidence	Implications
**1A**	Risk/benefit clear	Randomized controlled trials (RCTs) without important limitations	Strong recommendation, can apply to most patients in most circumstances without reservation

**1 B**	Risk/benefit clear	RCTs with important limitations (inconsistent results, methodological flaws)	Strong recommendations, likely to apply to most patients

**1 C+**	Risk/benefit clear	No RCTs but RCT results can be unequivocally extrapolated, or overwhelming evidence from observational studies	Strong recommendation, can apply to most patients in most circumstances

**1 C**	Risk/benefit clear	Observational studies	Intermediate strength recommendation; may change when stronger evidence available

**2A**	Risk/benefit unclear	RCTs without important limitations	Intermediate strength recommendation, best action may differ depending on circumstances or patients' or societal values

**2 B**	Risk/benefit unclear	RCTs with important limitations (inconsistent results, methodological flaws)	Weak recommendation, alternative approaches likely to be better for some patients under some circumstances

**2 C**	Risk/benefit unclear	Observational studies	Very weak recommendations; other alternatives may be equally reasonable

The coordinators (FL, PM) merged the committee preliminary statements with the observations and recommendations from the panel, and had the responsibility of summarizing the discussion on standards of treatment for OLCC that are presented in this manuscript.

## Results

### Loop colostomy (C) with staged procedure vs Hartmann's procedure (HP)

Loop colostomy is a historical component of the staged therapeutic schema for OLCC. During the first stage, the obstruction is managed by the colostomy. The second stage takes place a few weeks later when the tumour is resected and the colostomy is closed (two stage procedure) or, alternatively, the colostomy can be closed at a third stage. There is only one RCT study, by Kromborg et al in 1995, comparing emergency colostomy with three stages procedure (58 patients) versus HP (63 patients) for OLCC. The authors showed no difference in terms of mortality (8/58 vs. 8/63 patients) and morbidity rate, recurrence rate and cancer specific survival; the overall length of hospital stay was shorter in the resection group [9]. However this RCT has some important limitations due to methodological flaws: no prior sample size estimation; a 15-year accrual period; procedures being performed by 36 attending and training surgeons; incomplete follow up; heterogeneous underlying pathology (with non-malignant strictures accounting for 14% of cases).

Previously Fielding et al. in 1979 published a prospective non-randomised study (PNRS) which showed the same mortality rate for both groups [10]; however the study was affected by strong bias selection. A Cochrane systematic review in 2008 by De Salvo rt al, compared staged procedure vs. primary resection, and found similar mortality with either strategy [11]. It should be noted that the Kronborg study was excluded for methodological weaknesses. In theory, several benefits might be associated with creation of a loop colostomy: it provides colonic decompression; minimizes surgical trauma; reduces the risk of contamination from unprepared bowel; allows staging and multidisciplinary evaluation prior to definitive treatment.

Our literature review reveals that C does not provide any short- or long-term benefit over the HP whereas the multiple operations are associated with longer overall hospital stay: 49 days in group C vs. 35 days in HP group (p = 0.01); finally the staged approach shows a not significant tendency to expose the patient to a higher cumulative morbidity as a result of multiple operations[9].

*Recommendation:*HP should be preferred to C for OLCC, since C appears to be associated with longer overall hospital stay and need for multiple operations but not with a reduction in peri-operative morbidity (Grade of recommendation 2B).

*Advice:*the role of staged procedure, with preference at the two stages operation, should be considered (a) in a clinical situation where a surgical approach like "damage control" could be applied as happens in trauma scenario (b) when neoadjuvant multimodality therapy can be expected, or c) unresectable disease.

### Hartmann's procedure (HP) vs. primary resection and anastomosis (PRA)

There are no RCTs comparing HP and PRA; thus neither grade A and B evidence are available.

In 2004 Meyer et al by a prospective non randomized multicenter study compared, in emergency scenario, 213 patients undergoing HP to 340 patients undergoing PRA for OLCC. The mortality rate in the case of palliation for HP and PRA respectively was 33% vs. 39% and in case of curative intent for HP and PRA respectively 7,5% vs. 9,2%, however both of them without statistical difference; also the morbidity rate was not significantly different among groups; finally the HP was the most frequent surgical option [6]. The authors made a substantial effort in planning the study, collecting and analyzing data, however the number of participating institutions was very high (309) and heterogeneous spanning from regional to university hospitals. Finally among prospective non randomized and retrospective studies the rates of anastomotic leak in patients with OLCC treated with PRA range from 2,2% to 12% [5,6,12-14], which are similar to those reported for elective surgery ranging from 1,9% to 8% [15-18].

Furthermore our literature review suggests that HP might be associated with worse long-term outcomes. Villar et al. in 2005 published a prospective non randomized study comparing HP in 20 patients to PRA in 35 patients divided into ICI/SC or TC: they reported 5-year overall survivals of 38% and 41-45% for HP and PRA (divided into subgroups) respectively; however this difference was likely the result of selection bias as anastomosis was likely avoided in higher-risk patients [12,14].

The absence of anastomosis makes HP a technically easier operation and obviously eliminates the risk of colon dehiscence in a already complex scenario such as occurs in high grade obstruction: thus HP still remains an option also suitable by less experienced and non-specialist surgeons. The main disadvantages of HP is clearly the need for a second major operation to reverse the colostomy, which will be also associated with a risk of anastomotic dehiscence similar to PRA. Furthermore, it is somewhat disappointing to observe that the stoma reversal rate is only 20% in those patients with colon cancer [12,19]. PRA offers the advantages of a definite procedure without need for further surgery. Its main disadvantages are related to the increased technical challenge and to the potential higher risk of anastomotic leakage that occurs in the emergency setting.

Although PRA appears, at least in theory, more appealing than HP in OLCC, several parameters (patient and surgeon related) should be taken in consideration prior to choose the surgical procedure [5,14,20].

Risk stratification is at the base of patient selection. The Association of Coloproctology of Great Britain and Ireland (ACPGBI) study of large bowel obstruction caused by colorectal cancer identified four important predictors of outcome - age, ASA grade, operative urgency, and Dukes' stage [5]. Similar results were shown by other studies [14,20]. Recent large studies demonstrated that mortality rate after PRA of obstructive right colon cancer is higher than mortality after PRA for OLCC [5,14,21], whereas one study did not show any difference [22]. This findings could be explained by the fact that almost all patients with right-sided obstruction are treated by one stage resection and anastomosis, whereas patients with OLCC are carefully selected according to risk.

Keeping in mind these considerations the HP could be appropriate for patients deemed to be at high risk. Moreover the same considerations could explain the results of a questionnaire survey of American Gastrointestinal Surgeons in 2001 who responded that 67% would perform HP and 26% a simple colostomy in the high-risk patient [23]. Otherwise we should assume a lack of adherence to the literature evidence in the clinical practice or difficulty in changing from surgical tradition.

The experience and subspecialty of surgeon seems to be a primary factor in the choice of anastomosis or end colostomy. It has been shown that primary anastomosis is more likely to be performed by colorectal consultants rather than general surgeons, and by consultants rather than unsupervised trainees [20]. The ACPGBI study has shown that the mortality rate following surgery was similar between ACPGBI and non-ACPGBI members [5]. This result can be challenged as the study was done on a voluntary basis. The Large Bowel Cancer Project showed that registrars had a higher mortality rate than consultants after primary resection for obstruction in the late 1970 s, and this result has remained unchanged 20 years later in the Zorcolo study [1,20]. Other studies have also shown that unsupervised trainees had significantly greater morbidity, mortality and anastomotic dehiscence rates [10,24].

*Recommendation:*HP offers no overall survival benefit compared to segmental colonic resection with primary anastomosis in OLCC (Grade of recommendation 2C+); HP should be considered in patients with high surgical risk (Grade of recommendation 2C)

### Primary resection and anastomosis (PRA): total or subtotal colectomy (TC) vs. segmental colectomy (SC)

There is only one RCT, write out SCOTIA study group (Subtotal Colectomy versus on Table Irrigation and Anastomosis) in 1995, that compared the TC (47 patients) vs. SC (44 patients) and ICI. There were no differences in mortality, overall morbidity and rates of single complications (superficial and deep surgical site infections, anastomotic leakage). In regard of long-term outcomes, patients undergoing TC were noted to have a statistically higher number of daily bowel movements compared to ICI/SC. The authors concluded that SC following ICI should be therefore preferred to TC [25].

Another non-randomised study comparing the two techniques did not show any difference in mortality but showed significantly more surgical postoperative complications in the ICI group and in particular superficial surgical site infections [26].

TC as a one-stage resection anastomosis in OLCC allows the surgeon to encompass a massively distended and faecal-loaded colon [27,28]; moreover the proximal colon dilatation makes difficult the detection of synchronous cancer and so TC could *bypass *the need for further operation especially in severely ill patients. However we can't extend the use of TC as a prophylaxis of future malignancy outside hereditary tumours syndromes [27].

In the 1980 s, segmental colectomy with ICI was suggested as an alternative operation. It has the benefit of making an anastomosis on a prepared bowel and preserving the normal colon. The main concerns are the prolonged operative time, the risk of spillage and contamination, and the need for increased expertise[25].

Absolute indications for STC in OLCC are right colon ischemia, cecal serosa tears or perforation, and synchronous proximal malignant tumours which occur in 3 to 10% of cases [27]; it is a one stage radical oncological resection with advantages to treat synchronous proximal tumours, prevent metachronous cancer, to avoid stoma creation and to remove the colon as a septic content; but the major disadvantages are resection of healthy colon, resulting in poor functional results with many patients complaining of diarrhoea afterwards [25,27,28].

*Recommendation:*TC for OLCC (without cecal perforation or evidence of synchronous right colonic cancers) should not longer be preferred to SC with ICI, since the two procedures are associated with same mortality/morbidity, while TC is associated with higher rates impaired bowel function (Grade of recommendation 1A).

### Primary resection and anastomosis (PRA): Segmental colectomy (SC) with intraoperative colonic irrigation (ICI) vs. Segmental colectomy (SC) with manual decompression (MD)

Lim et al in 2005 published the only RCT comparing ICI (24 patients) with MD (25 patients) in OLCC. They concluded that MD is a shorter and simpler procedure than ICI, and offers similar results in terms of mortality, morbidity or anastomotic leak rates, but the study was underpowered [29].

On average, the ICI increases duration of surgery by an hour, although this time can improve with increasing experience. To overcome the problems of ICI, various studies suggested segmental resection and primary anastomosis with MD only, as an safe alternative [29-32]. This idea was supported by various RCTs comparing mechanical bowel preparation, with no preparation in elective open colonic surgery.

The results were separately examined in a Cochrane systematic review of 9 RCTs [15] and in a metaanalysis of 7 RCTs [33]. Both studies concluded that there is no convincing evidence that mechanical bowel preparation is associated with reduced rates of anastomotic leakage after elective colorectal surgery.

Finally in 2009 Kam et al published a systematic review on ICI vs. MD in left-sided colorectal emergencies: they included 1 RCT, 1 prospective comparative trial and 5 prospective descriptive case series and concluded that, although the power of studies is poor and large-scale prospective randomized trial is desirable, no statistical significance could be shown between the two procedures [34].

*Recommendation:*during segmental resection and primary anastomosis for OLCC (without cecal perforation or evidence of synchronous right colonic cancers), either MD or ICI can be performed as the two techniques are associated with same mortality/morbidity rate. The only significant difference is that MD is a shorter and simpler procedure. Either procedure could be performed, depending of the experience/preference of the surgeon (Grade of recommendation 1A).

### Endoscopic Colonic Stents (SEMS)

Colonic stents were introduced in the 1990 s and have been used for palliation or as a bridge to surgery: following release of the obstruction with an endoscopic stent the patient is properly staged and offered multidisciplinary treatment and eventually elective or semi-elective surgery [35].

#### A) Palliation: endoscopic colonic stents (SEMS) vs. colostomy (C)

There are three RCTs comparing colostomy vs. SEMS for palliation of malignant colonic obstruction [36-38].

Xinopulos et al in 2004 randomized 30 patients. In the SEMS group placement of the stent was achieved in 93.3% (14/15 pt); there was no mortality. In 57% (8/14) of patients in which the stent was successfully placed, colonic obstruction was permanently released (i.e. until death). Mean survival was 21,4 month in SEMS group and 20,9 months in C group. Mean hospital stay was quite high in both groups and significantly higher in group C: 28 days vs. 60 days. This study presented several limitations, and the small sample size might have limited the ability to discern differences between groups [36]

Fiori et al in 2004 randomized 22 patients to either C or SEMS: mortality was 0% in both groups, morbidity was similar. SEMS group had shorter time to oral intake, restoration of bowel function, and hospital stay. This study was also limited by the small simple size and by the lack of follow up [37]

The Dutch Stent-in I multicenter RCT was planned to randomized patients with incurable colorectal cancer to SEMS or surgery: the study was terminated prematurely after enrolling 21 patients because four stent-related delayed perforations resulting in three deaths among 10 patients in the SEMS group. There are no clear explanation for such a high perforation rate; the authors pointed out that limited safety data existed fort he stent used in their study (WallFlex, Boston Scientific Natick, MA) [38]. Indeed, subsequent studies of Wallflex stent for colonic obstruction reported a perforation rate of about 5% [39-42] which is in line with what commonly observed with other stents [42].

The feasibility, safety, and efficacy of SEMS have been analyzed by retrospective studies. There are four systematic reviews analysing the outcome of SEMS for large bowel obstruction with the Sebastian study being the most complete and focused one [43-46]. He retrieved 54 studies with a total of 1198 patients and the median rates were: technical success 94%, the clinical success 91%, the colonic perforation 3,76%, the stent migration 10%, the re-obstruction 10%, stent-related mortality 1% [44]. These studies have shown that colonic stenting is a relatively safe technique with high success rates.

The influence of colonic stents on oncologic outcomes has been questioned but no exhaustive answer is available. Indeed, several studies suggested that primary tumour resection with palliative intent, would prolong survival in patients with stage IV colorectal cancer [47,48]. However the power of these retrospective studies is poor due to the study design, no uniform adjuvant therapies among groups, and the bias to compare unresectable stage IV cancer patients with resectable stage IV cancer patients.

On the other hand, several comparative, retrospective studies did not show any significant difference in term of overall survival after 3 and 5 years of follow up, between emergency surgery and stent placement [49,50].

Colonic stents have an attractive role in a multimodality approach to obstructive colon cancer; however close clinical observation is required: for example there is one literature report that colonic stent may increase the risk of colon perforation in patients who are candidates for bevacizumab: thus according to authors alternative treatments to SEMS in these patients should be considered [51].

*Recommendation:*in facilities with capability for stent placement, SEMS should be preferred to colostomy for palliation of OLCC since stent placement is associated with similar mortality/morbidity rates and shorter hospital stay (Grade of recommendation 2B).

*Advice:*authors cautiously suggest to consider alternative treatments to stent in patients eligible for further bevacizumab-based therapy

#### B) Bridge to surgery: endoscopic colonic stents and planned surgery vs. emergency surgery

Cheung et al. recently published a RCT comparing endolaparoscopic approach (24 pts) *vs*. conventional open surgery (24 pts). In patients who were randomized to the endolaparoscopic group, an SEMS placement for colon decompression was attempted within 24-30 hours from admission and an elective laparoscopic-assisted colectomy was performed within two weeks following SEMS placement. Patients who were randomized to the open surgery group underwent emergency HP or TC with ICI on the same day of admission. Over a 3-years period, 50 patients were enrolled and 48 were available for the final analysis (24 in the open surgery group and 24 in the endolaparoscopic group). Overall, only 6 of11 patients undergo HP had subsequent reversal; PRA was conducted in 13 patients all but two without covering stoma; two patients experienced anastomotic leak (2 out of 11, 18,8%) requiring end colostomy and one of these had subsequent reversal; thus 1-stage operation was performed successfully in 38% and 75% avoided a permanent colostomy. Colon decompression by SEMS was achieved in 83% of patients while the 17% had HP At the time of planned surgery, 67% of patients in the endolaparoscopic group had successful 1-stage operations performed and the 4 remaining patients had diverting ileostomy (33%); finally in the endolaparoscopic group no one was given a permanent stoma. Furthermore, patients randomized to the endolaparoscopic group compared to emergency surgery had significantly greater successful 1-stage operation (16 vs.9; p = 0,04), less cumulative blood loss (50 ml vs. 200; p = 0,01), less wound infection (2 vs. 8; p = 0,04), reduced incidence of anastomotic leak (0 vs.2; p = 0,045), and greater lymph-node harvest (23 vs.11; p = 0,05).

Cheung and colleagues suggest that colon decompression provides time for resuscitation, adequate staging, bowel preparation and safer, minimally-invasive elective resection. Indeed, the rate of primary anastomosis is twice that following emergent surgery, and the stoma rate and the postoperative complications are significantly reduced [52].

Observational studies comparing SEMS followed by planned surgery with emergency surgery (HP, or PRA). Martinez-Santos in a prospective non-randomised study comparing 43 patients in the SEMS group with 29 patients in emergency surgery group reports a 95% technical success rate of SEMS; however only 26 patient in the SEMS group had a further surgical operation: at the time of planned surgery for SEMS the comparison of median rate between SEMS vs. emergency surgery shows: primary anastomosis was 84,6% vs. 41,4% with p = 0,0025; morbidity was 40% vs.62% p = 0,054; ICU stay was 0,3 vs.2,9 days p = 0,015; reintervention was 0% vs. 17% p = 0,014; mortality was 9% vs. 24% however without reaching statistical significance [53]. However the study is somewhat confusing because it include also a large population of palliative SEMS (14) and the two population in SEMS are sometime mixed and then compared to emergency surgery group. Similar results are reported also in less robust retrospective studies [50,54].

Tinley in 2007 performed a meta-analysis of non-randomised studies that compared SEMS and open surgery for malignant large bowel obstruction: SEMS was attempted in 244 out of 451 patients (54,1%) with a success rate of 92,6%; mortality occurred in 14 (5,7%) in SEMS and in 25 (12,1%; p = 0,03) in emergency surgery [55]. This metaanalysis however was likely impaired by the heterogeneity of studies, since both patients stented for palliation or as a bridge to surgery were included. In this meta-analysis mortality rate for stenting (5.7%) was much higher than the 0.6% rate reported in a large systematic review [45]

Little is known on oncologic outcomes of using SEMS as a bridge to elective surgery. A recent paper recommended that surgery should be scheduled shortly after stent insertion because the risk of tumour seeding from perforation and dislocation of stent [56]. However selection bias of indication and timing of stenting could explain the high level of complications reported with SEMS and consequently the advice of authors regarding long-term survival [57]. Finally there is no study available comparing survival in SEMS versus other surgical options.

The cost effectiveness of SEMS is an important parameter as stents are very expensive. It is thought that their cost is offset by the shorter hospital stay and the lower rate of colostomy formation. Two decision analysis studies from the US and Canada calculated the cost-effectiveness of two competing strategies - colonic stent versus emergency primary resection for OLCC [58,59] Both concluded that colonic stent followed by elective surgery is more effective and cost efficient than emergency surgery. A small retrospective study from the UK in 1998 showed that palliative stenting compared to surgical decompression allows saving a mean of £1769, whereas the stenting as a bridge to elective resection vs. emergency HP followed by elective reversal saved a mean of £685 [60]. A RCT from Greece comparing SEMS and colostomy for palliation of patients with inoperable malignant partial colonic obstruction showed very small difference in the costs, with the stent group being 6.9% (132 euros) more expensive per patient [36]. Another study from Switzerland reported SEMS to be 19.7% less costly than surgery [61]. None of these studies incorporated the hidden costs of stoma bags used in the community. Although stents seem to be cost effective, results are difficult to compare because costs calculations vary in different health care systems, costs differ for palliation and bridge to surgery, and the cost of stents is likely to decrease over time.

*Recommendation:*SEMS should be used as a bridge to elective surgery in referral centre hospitals with specific expertise and in selected patients mainly as their use seems associated with lower mortality rate, shorter hospital stay, and a lower colostomy formation rate (Grade of recommendation 1B).

## Conclusions

This consensus conference aimed to analyze the available scientific evidence on treatment modalities for OLCC and how this is implemented in clinical practice. The goal of the authors was to offer practical and scientifically supported suggestion to manage OLCC.

The committee made every effort to collect and classify the best available scientific evidence on treatment of OLCC (Table 2). Subsequently, the audit and panel discussion played a pivotal role in the statement declarations.

**Table 2:** Evidences used for the present Consensus Conference

Evidence type	C **vs**. HP	HP **vs**. PRA	TC **vs**. SC	SC+ICI **vs**. SC+MD	SEMS **vs**. C in palliation	SEMS + surgery **vs**. surgery	Total of studies
RCT	1 [9]	0	1 [25]	1 [29]	3 [36-38]	1 [52]	9

PNRS/OS	1 [10]	6 [5,6,12-14,23]	1 [26]	3 [30-32]	0	3 [50,53,54]	14

CSR	1 [11]	0	0	0	0	0	1

SR	0	0	0	1 [34]	4 [43-46]	0	5

MA	0	0	0	0	0	1 [55]	1

Cost analysis	0	0	0	0	0	5 [36,58-61]	5

[references]

All the participants at consensus conference agree that the literature power is relatively poor and the existing RCT are often not sufficiently robust in design thus, among 6 possible treatment modalities, only 2 reached the Grade A.

To help in decision making the authors wish to suggest surgeons to consider 3 further key points approaching OLCC: patient stratification according to the ACPGBI rules; clinical environment; surgeon skill.

The target as usual is to offer the best option for the patient; starting from this point of view also historical surgical option could still play a valid role. The staged procedure, with preference to the two stages, should be reserved when multimodality therapy is expected or in case of "dramatic" scenarios.

PRA with manual decompression is a safe option and appears to be associated with best outcomes. HP might still have a role in patients at high risk for anastomotic dehiscence. TC is an appealing option in case of synchronous polyps or cancer and/or impending or actual perforation of the right colon. SEMS represent a valuable option both for palliation and as a bridge to elective surgery. Obviously high clinical and technical expertise is mandatory to safely and successfully treat colonic obstruction by stents: due to this consideration routine use in practice is still limited.

However we strongly support a judicious application of the procedure and encourage increased use of stents after adequate training in referral hospitals with a goal of further testing this modality.

## List of abbreviations

ACCP: American College of Chest Physician; ACPGBI: Association of Coloproctology of Great Britain and Ireland; CSR: Cochrane Systematic Review; SEMS: Endoscopic Colonic Stenting; HP: Hartmann's Procedure; ICI: Intra-operative Colonic Irrigation; C: Loop Colostomy; MD: Manual Decompression; MA: MetaAnalysis; OS: Observational Study; OLCC: Obstructed Left Colon Cancer; PRA: Primary Resection and Anastomosis; PNRS: Prospective Non-Randomized Study; RCT: Randomized Controlled Trial; SC: Segmental Colectomy; SR: Systematic Review; TC: Total/Subtotal Colectomy.

## Competing interests

The authors declare that they have no competing interests.

## Authors' contributions

**LA: **conception and design of the study; organiser of the consensus conference; preparation of the draft; he merged the committee preliminary statements with the observations and recommendations from the panel, he summarised the discussion on standards of treatment for OLCC; manuscript preparation and review. FC: conception and design of the study; organiser of the consensus conference; manuscript review. SDS: manuscript review. BF, CV, LA, RA, TJJ: preparation of the draft inclusive of preliminary statements; manuscript review. PAD: conception of the study; organiser of the consensus conference; main contributor to critical discussion of the draft. ARE, SPH, JH, MEE: main contributors to critical discussion of the draft, manuscript review. FL: preparation of the draft inclusive of preliminary statements. He merged the committee preliminary statements with the observations and recommendations from the panel, he summarized the discussion on standards of treatment for OLCC. MP: he merged the committee preliminary statements with the observations and recommendations from the panel, he summarized the discussion on standards of treatment for OLCC; manuscript preparation and review. All Authors read and approved the final manuscript.
